# Identification and Categorization of the Distinct Purposes Underpinning the Use of Digital Health Care Self-Monitoring: Qualitative Study of Stakeholders in the Health Care Ecosystem

**DOI:** 10.2196/58264

**Published:** 2025-04-03

**Authors:** Mattias Elg, Daan Kabel, Ida Gremyr, Jesper Olsson, Jason Martin, Frida Smith

**Affiliations:** 1 Department of Management and Engineering Linköping University Linköping Sweden; 2 Department of Technology Management and Economics Chalmers University of Technology Gothenburg Sweden; 3 Swedish Medtech Stockholm Sweden

**Keywords:** self-monitoring, healthcare provider, medtech provider, digital health, healthcare ecosystem, qualitative study, technology, semi structured interview, macro perspective, telehealth, telemonitoring

## Abstract

**Background:**

Digital health care self-monitoring has gained prominence as a tool to address various challenges in health care, including patient autonomy, data-informed decision-making, and organizational improvements. However, integrating self-monitoring solutions across a diverse ecosystem of stakeholders—patients, health care providers, policy makers, and industry—can be complicated by differing priorities and needs.

**Objective:**

This study aimed to identify and categorize the distinct purposes underpinning the use of digital health care self-monitoring. By mapping these purposes, the research seeks to clarify how technology design and implementation can be better aligned with stakeholder expectations, thereby enhancing adoption and impact.

**Methods:**

A qualitative design was used, drawing on 31 in-depth, semistructured interviews conducted with stakeholders in the Swedish health care ecosystem. Participants included patients, advocacy groups, health care professionals, policy makers, pharmaceutical representatives, and technology developers. Data were analyzed thematically using an inductive coding approach supported by NVivo 12 (Lumivero). Emerging themes were refined through iterative discussion among the research team and validated by presentation to health care practitioners.

**Results:**

A total of 8 distinct purposes of digital health care self-monitoring emerged: (1) emancipate (enhance patient autonomy), (2) learn (understand health behaviors), (3) improve (enhance patient health), (4) engage (bolster patient involvement), (5) control (manage adherence and symptoms), (6) evaluate (assess health parameters), (7) innovate (advance interventions and processes), and (8) generate (drive new initiatives). These purposes form three categories of value creation: (1) improving the patient-provider link, (2) leveraging big data analytics for knowledge creation, and (3) using digital infrastructure to develop new care processes.

**Conclusions:**

Our findings demonstrate that digital health care self-monitoring serves multifaceted aims, ranging from individual patient empowerment to ecosystem-wide innovation. Designing and implementing these tools with an explicit understanding of all stakeholders’ “why” can help address potential conflicts (eg, balancing patient autonomy with clinical control) and facilitate more holistic adoption. Ultimately, this study underscores the importance of clear, purpose-driven approaches to promote better health outcomes, knowledge generation, and care process improvements.

## Introduction

Health care is witnessing a discernible push toward the integration of digital medical and health-related technologies [1]. Many believe that this integration is central to managing critical challenges of access to care, enhancing the quality of medical services, reducing health care costs, and personalizing patient care. The shift is affected by and affects a wide range of stakeholders within the health care ecosystem, including policy makers, health tech developers, and patients who want to live a more independent and active life.

Terms such as “Telemonitoring,” “e-health,” and “Medicine 2.0” have emerged [1,2], capturing a range of innovations from digital health apps through health information systems to wearable biosensors that facilitate remote health monitoring [3]. As these technologies gain prominence, they support varied purposes and present potential impacts on patient care, health data management, and broader health practices [3]. While existing literature has shed light on specific areas, such as evaluations of distinct tools [4,5], their impact on patient outcomes [6,7], and their integration into care pathways [8,9], the realization of these tools is far from straightforward. For instance, medical technology firms tend to drive a technology push, focusing on innovative solutions. Clinical researchers are interested in leveraging these technologies for robust data collection, which can inform future health care practices, for example, as a tool to support healthy lifestyles through behavioral change. On the other hand, patients seek tools that empower them with more information and control over their health, aligning technology with their day-to-day needs. One such tool is digital health care self-monitoring, which, in combination with other health interventions, has the potential to affect behavioral change and promote healthy lifestyles, for example, in weight loss treatments [10] and physical activity [11]. This study focuses on digital health care self-monitoring as it is a fairly accessible technique with potential benefits for health care providers, as well as patients. Thus, various purposes would be beneficial to account for when a digital tool is designed and developed. Thus, it is crucial to understand that the purposes of realizing digital technology may vary between stakeholders.

Addressing this issue is crucial for developing a comprehensive understanding of digital health, particularly in the context of digital health care self-monitoring [12]. First, understanding the purposes not only offers insight into anticipated outcomes but also directly informs how self-monitoring tools ought to be designed to achieve these outcomes [13,14]. Second, this comprehension provides a basis for evaluation, allowing for assessments of whether the tool meets its objectives [13]. Third, an awareness of the “why” can help identify specific operational or user-related challenges, thereby enabling more targeted solutions [15]. Fourth, as health literacy becomes a priority for organizations to adhere to, emphasizing patient-centric approaches and recognizing diverse objectives can promote greater acceptance and usage among patients [16]. In other words, greater acceptance is linked to patients’ understanding of the technology’s broader goals in enhancing their health literacy [17]. Finally, grasping the underlying reasons can shed light on potential cultural and ethical dimensions, promoting a more holistic and responsible approach to implementation [18].

In our definition of digital health care self-monitoring, 3 pivotal components are central. First, the “digital” component suggests the use of technologies such as smartphones, computers, and wearable devices. These tools aim to enable real-time data collection, storage, and analysis. Second, the “health care” facet underscores that self-monitoring is anchored within the context of health care delivery. This means it is often prescribed or recommended to patients by health care professionals, making it an integral part of already existing care. Third, the component of “self-monitoring” is central to our definition. It encompasses two dimensions: (1) awareness of bodily symptoms, sensations, daily activities, and cognitive processes, and (2) measurements, recordings, and observations that inform cognition or provide insights for independent action or consultation with care providers [19]. In sum, the aim of this paper is to explore the different purposes of digital health care self-monitoring as a means to guide its design and development to best support its range of stakeholders.

## Methods

### Research Design and Approach

The importance of digital health care self-monitoring tools stems not only from their intrinsic characteristics but also from their active interplay within particular settings. It is here, drawing inspiration from the sociologist Bruno Latour’s distinction between ostensive and performative definitions, that a deeper understanding can be gained [20] rather than just drawing attention to the static, inherent attributes of these tools—the ostensive aspect—our focus shifts to the performative, understanding how they weave into and function within the broader health care ecosystems. Recognizing the multifaceted nature of these tools, it becomes clear that an empirical approach, in line with Latour’s emphasis on the performative, is necessary in order to capture the motivations, implications, and nuances that shape the use of digital health care self-monitoring. Thus, exploring the “why” behind digital health care self-monitoring should go beyond a mere understanding of its visible and immediate features. This exploration requires an empirical examination of the perspectives of a diverse set of stakeholders shaping its design and use, encompassing patients, professionals, health care administrators, developers, IT experts, tech industry representatives, and macrolevel policy actors [18]. Such an exploration lends itself to a qualitative research design based on an inductive approach and data from an interview study. The context of this study is the Swedish health care system, the system that all researchers have interacted with through either professional health care work (FS and JO) or as researchers (all authors). The 6 researchers involved in this study (2 female and 4 male) all have experience in qualitative research, spanning from 5 to over 25 years. All researchers besides DK hold a PhD. Furthermore, the researchers have various backgrounds relevant to the study of digital tools in a health care setting: 3 (ME, DK, and IG) have an engineering background, 2 (FS and JO) have a background as registered nurses, and 1 (JM) a human resource management background.

### Participant Selection

A purposive sampling technique was used to select the 31 participants; the inclusion criteria were to (1) have direct experience or involvement with digital health care self-monitoring technology, (2) a minimum of 5 years of professional experience, and (3) working in Sweden (ensuring experience from the same health care system). All potential participants approached agreed to be interviewed.

In our research, we relied on 2 distinct datasets to gather comprehensive insights on the subject of digital health care self-monitoring. The first dataset encompassed 20 interview respondents selected from a wide spectrum of the health care ecosystem. These respondents were not just randomly chosen; rather, they represent a diverse cross-section of stakeholders involved in the implementation of digital health care self-monitoring. Among them were representatives from policy-making entities, patients (advocacy groups), multinational pharmaceutical companies, and tech firms specializing in health solutions. Furthermore, we had IT managers who oversaw technological integrations within health care systems, as well as administrators and developers responsible for the national health care system’s digital architecture. We also involved representatives from clinical data registries to support a more inclusive and balanced perspective. Respondents were identified through 2 main strategies. First, through our existing network with individuals working with digital self-monitoring and MedTech. Second, we reached out to a representative from the Swedish Association of Local Authorities and Regions at the national level. Interviews in this dataset were performed in the first half of 2022.

Our second dataset was more focused on its scope but equally important to our study. It comprised 11 interviewees directly involved in a pilot study concerning digital self-monitoring in cancer care. This group provided insights as they were deeply embedded in an implementation process. Their perspectives, derived from hands-on experiences, were instrumental in shedding light on the views of health care professionals, software developers, and administrative managers directly participating in such pilot programs. In this sample, our goal was to understand the practical details and operational aspects of incorporating digital self-monitoring tools into a specific health care area such as cancer care. Interviews in this dataset were performed in the second half of 2023.

### Data Collection

We conducted 31 in-depth interviews (see Table 1, interviews conducted by ME, JO, DK, and JM); interviews were recorded and transcribed (anonymized). Each interview was carried out by 2 interviewees and lasted between 30 and 60 minutes; and was conducted either face-to-face (in a workplace) or through video conferencing, depending on the preference and availability of the participant. The interviews were semistructured, using an interview guide (Multimedia Appendix 1) that comprised open-ended questions designed to elicit detailed responses regarding the participant’s experiences, perspectives, and insights on digital health care self-monitoring.

For these 2 distinct datasets, 2 interview protocols were designed and used. The interview protocols included, first, a set of general questions that all 31 participants answered, and second, specific questions depending on the participants’ micro (I1-I6) or macro (I7-I31) roles and perspectives on the use of digital health care self-monitoring tools. Examples of the questions in the interview protocol include “How do you view self-monitoring in the healthcare system?” “What is the greatest benefit of self-monitoring, and what are your key takeaways?” and “What challenges do you see in implementing self-monitoring?” These general questions allowed the interviews to take different directions based on the participants’ own views. The 2 interview protocols differed in terms of the set of specific questions and the participants’ roles. Microlevel perspectives refer to the purposes health care professionals or advocacy groups (I1-I6) have in relation to digital health care self-monitoring. Examples of specific questions include “How was self-monitoring perceived and experienced by your patients?” and “How have you used the information from self-monitoring to assist your patients?” Macrolevel perspectives refer to why policy-making entities, pharmaceutical companies, tech firms, IT managers, software developers, and administrative managers encourage or could benefit from self-monitoring tools (I7-I31).

Together, the combined insights from these 2 datasets provided us with both a macro and micro perspective on the digital health care self-monitoring ecosystem. The data collection ended when new data did not lead to any additional identified purposes for digital health care self-monitoring; that is, it did not add further to the aim of the study [21]. Furthermore, the rather narrow focus of the study points to the fact that the number of interviews (n=31) is sufficient to reach saturation [22].

**Table 1:** Interviewees, their roles, and associated stakeholders.

Identifier	Stakeholder	Role	Gender	Age (years)
I1	Patient and advocacy group	Organize self-monitoring campaigns and patient education programs.	Woman	53
I2	Advocacy group	Organize and conduct research on self-monitoring.	Woman	47
I3	Health care professional, cancer care	Reads and responds to self-monitored data from patients and provides health care services.	Woman	55
I4	Health care professional, cancer care	Reads and responds to self-monitored data from patients and provides health care services.	Woman	51
I5	Health care professional, cancer care	Reads and responds to self-monitored data from patients and provides health care services.	Woman	53
I6	Health care professional, cancer care	Reads and responds to self-monitored data from patients and provides health care services.	Woman	50
I7	Technology firm specializing in health solutions	Provision of self-monitoring solutions to support clinical decision-making.	Man	53
I8	Technology firm specializing in health solutions	Provision of self-monitoring solutions to support clinical decision-making.	Man	39
I9	Technology firm specializing in health solutions	Provision of self-monitoring solutions to support clinical decision-making.	Man	46
I10	Technology firm specializing in health solutions	Provision of self-monitoring solutions to support clinical decision-making.	Man	52
I11	Technology firm specializing in health solutions	Provision of self-monitoring solutions to support clinical decision-making.	Man	56
I12	Technology firm specializing in health solutions	Provision of self-monitoring solutions to support clinical decision-making.	Woman	54
I13	Technology firm specializing in health solutions	Provision of self-monitoring solutions and integration within the health care ecosystem.	Man	55
I14	Technology firm specializing in health solutions	Provision of self-monitoring solutions to support clinical decision-making.	Woman	54
I15	Technology firm specializing in health solutions	Provision of self-monitoring solutions to support clinical decision-making.	Woman	48
I16	Technology firm specializing in health solutions	Provision of self-monitoring solutions to support clinical decision-making.	Man	59
I17	Pharmaceutical company	Deliver medication on time based on self-monitored data.	Man	—^a^
I18	Pharmaceutical company	Deliver medication on time based on self-monitored data.	Man	44
I19	Pharmaceutical company	Deliver medication on time based on self-monitored data.	Woman	56
I20	IT manager, health care systems	Maintains the digital infrastructure of the hospital.	Man	63
I21	IT manager, health care systems	Maintains the digital infrastructure of the hospital.	Man	49
I22	IT manager, health care systems	Maintains the digital infrastructure of the hospital.	Woman	33
I23	Software developer, cancer care	Organizing and planning of health care services.	Woman	39
I24	Administrative manager, cancer care	Financing and planning for the integration of self-monitoring solutions.	Woman	67
I25	The national health care system’s digital architecture	Responsible for coordinating and modernizing the digital infrastructure of hospitals.	Man	48
I26	The national health care system’s digital architecture	Responsible for coordinating and modernizing the digital infrastructure of hospitals.	Man	48
I27	The national health care system’s digital architecture	Responsible for coordinating and modernizing the digital infrastructure of hospitals.	Man	71
I28	Policy-making entity	Develop and influence health care policies for self-monitoring.	Woman	—
I29	Policy-making entity	Develop and influence health care policies for self-monitoring.	Woman	61
I30	Policy-making entity	Develop and influence health care policies for self-monitoring.	Woman	74
I31	Policy-making entity	Develop and influence health care policies for self-monitoring.	Man	48

^a^Not available.

### Data Analysis

We conducted our analysis using a thematic approach [23]. An inductive coding method was adopted to explore and understand the experiences of our participants related to digital health care self-monitoring. This approach was selected as it emphasizes grasping the nuanced and central elements that participants encounter in their day-to-day use and interaction with digital health care self-monitoring tools. By focusing on their direct experiences, we aimed to uncover the underlying purposes of using digital health care self-monitoring. This ensured that the study was rooted in the real-world context of the participants. This approach is consistent with our performative stance, where the insights and perspectives of the actors within the ecosystem play a pivotal role in achieving a comprehensive and grounded understanding of the subject studied.

Each transcribed interview was read and coded by 1 researcher to identify emerging themes, which were later discussed and iterated with the other researchers. This thematic analysis unfolded on 2 levels. At the first level, we identified overarching themes that pinpointed specific purposes for using digital health care self-monitoring. The second level identified subthemes that highlighted variations and nuances, further elaborating and refining the primary themes from the first level. This analytical process was inherently iterative. As we progressed, 8 distinct purposes (or first-level themes) emerged successively. Respondent quotes have been used to elucidate the identified themes and subthemes in the Results section of this paper (to provide an overview, all quotes included in the paper are also found in Multimedia Appendix 2). While these quotes have been translated into English, it is crucial to acknowledge that translations can occasionally alter the original sentiment or subtlety. Nonetheless, we made every effort to preserve the true essence and intent of the quotes. For systematic analysis and theme generation, we used NVivo 12 (Lumivero) software. The analysis was validated in a presentation to about 15 health care practitioners. To further enhance trustworthiness, the researchers not directly involved in data collection (IG, JO, and FS) acted as external investigators [24].

### Ethical Considerations

The study does not fall under the domain of the Swedish Law on Ethics, including human subjects (2003:460), as no sensitive personal information was handled. In Sweden, approval from the national ethics board ethical review is needed when the research “entails the processing of sensitive personal data or personal data about violations of the law [or] involves a physical intervention conducted on a research participant” (p. 65) [25]. As no personal data was collected and no physical intervention was carried out, a formal application was not needed; still, the study was carried out in compliance with the ethical standards of the institution. However, all participants were informed about the purpose of the study and written informed consent to participate in the study was obtained. The participants were informed that their responses would be anonymized. The respondents did not receive any compensation for participation and did not have an established relationship with the interviewers.

## Results

The research identifies 8 distinct purposes: emancipate, learn, improve, engage, control, evaluate, innovate, and generate (Table 2). We categorize the purposes by starting with a focus on the patient, followed by ties to health care providers that also facilitate conditions for patient autonomy. As we move along this spectrum, the emphasis shifts to the more research-driven needs and means for improvements of health care systems’ deliveries. Extending further, a wider ecosystem emerges where various stakeholders harness the power of digital health care self-monitoring infrastructure for various initiatives. Each purpose sheds light on different aspects of the subject, mirroring the varied motivations and viewpoints of different stakeholders.

**Table 2:** The 8 purposes for using digital health care self-monitoring.

Purpose	Key questions
Emancipate (granting patients autonomy)	How can the technology support patients to gain a sense of autonomy and competence so that they feel secure?
Learn (understanding patient symptoms and behaviors)	What behaviors are related to better health outcomes? What are the normal values for the patient? How can the patient gain better self-awareness about the disease and its progression?
Improve (enhancing patient’s health)	How does the patient know that a change in treatment or lifestyle behavior is an improvement?
Engage (deepening patient involvement)	How can we involve patients more in their health?
Control (managing adherence and symptoms)	What is the health status of the individual patient? Does the patient adhere to the interventions?
Evaluate (assessing health parameters)	How well does a certain intervention (treatment, therapy, etc) work?
Innovate (improving interventions, health care processes, and technology)	How well does a certain intervention (treatment, therapy, etc) work? What new strategies can be incorporated into care delivery? How can technology streamline and innovate health care processes?
Generate (driving new initiatives)	How can existing digital infrastructure support new health initiatives?

### Emancipate

#### Overview

The purpose of “Emancipate” is to draw attention to how digital self-monitoring empowers patients to lead a life characterized by greater autonomy, even while maintaining a vital connection with their health care providers. This implies patients take active control over their health conditions, as data from self-monitoring provides patients with insights that they need to understand their health conditions better and to make choices that align with their personal health goals and values. This purpose is rooted in self-determination, as the competent patient—equipped with insights from tracking and a technology that bolsters relatedness—finds herself able to exercise increased autonomy in managing health. In addition, the 2 important aspects of the emancipation “purpose” are elaborated below.

#### Practicality

An advantage of digital self-monitoring lies in the immense practicality it offers. For patients, this technology negates the need for unnecessary physical visits to health care centers, especially when their health data fall within the normal range. This is captured in a statement by a representative from a technology association:

If [a patient] could just live [his or her] life without having to adapt to the opening hours and locations of healthcare, that’s what I desire. [you] want to live as normally as [you] can despite the illness, and this technology makes that feasible.

I10

#### Relatedness

Despite the shift towards increased patient independence, the feeling of connection and support from health care professionals remains invaluable. Self-monitoring may, therefore, imply a component of relatedness where patients find support in knowing that a safety net is always in place, with health care providers proactively monitoring their metrics and intervening when necessary. This is echoed in the words of a coordinator at the national level:

The team that manages my disease is always accessible in some way.

I30

Furthermore, this sentiment is reinforced by another statement:

The healthcare system always acts as a backup and proactively intervenes if health metrics start to change in a way that might necessitate treatment adjustments.

I13

An expressive illustration of this connected yet autonomous relationship comes from another quote:

She no longer feels the dread of death and doesn't always feel the need to be close to her healthcare centre.

I31

The “Emancipate” theme embodies the dual nature of digital self-monitoring: fostering patient autonomy while ensuring a strong and supportive bond with health care providers. Through the practical benefits of technology, patients can avoid unnecessary interruptions in their daily lives. Meanwhile, the present backing of health care professionals ensures patients never feel isolated. This balance transforms the health care journey into a collaborative endeavor, where the empowered patient, equipped with insights and support, charts their course towards better health.

### Learn

#### Overview

The “Learn” theme delves into the power of digital self-monitoring to offer insights into one’s health and well-being. With a focus on pattern discovery, signal detection, and flexible learning from tracking, patients are empowered to make informed decisions about their health. This exploration allows patients not only to take charge of their health but also to identify potential triggers or patterns related to their well-being. Furthermore, an emphasis on flexibility in the design of self-monitoring tools illuminates the idea that they can be tailored to meet the individual and evolving needs of patients.

#### Pattern Discovery

A vital component of self-monitoring is the identification of patterns that can provide actionable insights. A representative from the cancer foundation remarked:

...to rate their own sense of quality of life during treatment and then also... the idea was to take more control over one's daily life so that patterns related to what one ate or how one moved could be discerned. If one felt unwell one day, why? How did I live that day compared to days I felt well?

I2

#### Signal Detection

Identifying out-of-range health results and anomalies plays a crucial role in self-monitoring. It is pivotal in ensuring that individuals or systems can recognize and respond to certain cues or thresholds. Often, professionals such as clinicians define these threshold values to ensure accuracy and relevance. A representative from a technology company provided an illustrative example of this concept, remarking:

When a measurement exceeds a specific threshold, what kind of self-care recommendations should be provided?

I6

#### Learning in Interactions

The shared knowledge during interactions between patients and professionals can further illuminate patterns and offer insights. A statistical consultant in the registry business noted:

If you manage your diet, exercise, sleep well, and take your medication correctly... then you can see a certain pattern statistically. Then it becomes interesting to ask: 'How did you feel when you woke up this morning? How did you think your day would be compared to how your day went when you went to bed in the evening?

I26

#### Flexible Tracking Systems

Recognizing that not all patients have consistent needs or interests in tracking, the importance of flexibility is underscored. A patient shared:

I track when I have a specific question to answer. I believe in doing it for a short period... because when I feel good enough, I don't want to spend time tracking. I want to live my life...

I9

The “Learn” theme encapsulates the role of digital self-monitoring, enabling patients to identify patterns and signals in their understanding of disease and health behaviors. While pattern discovery allows for a better understanding of lifestyle impacts, signal detection offers real-time feedback. Furthermore, the flexibility offered by tracking systems ensures that monitoring aligns with the individual needs of the patient, making it a tool for specific queries rather than a constant chore. This approach thus strives to cultivate a more informed and engaged patient equipped to navigate their health journey effectively.

### Improve

#### Overview

The purpose of “Improve” underscores the essence of using digital self-monitoring as a tool for health enhancement. Rooted in the proactive stance of patients toward their health, this theme articulates the tangible improvements patients experience by using such technology. By leveraging pattern discovery and signal detection from the “Learn” theme, patients are empowered to enact changes in their life, be it symptom control, disease prevention, or shifts in lifestyle habits.

#### Lifestyle Habit Improvements

A central aspect of the “Improve” theme revolves around the positive changes in lifestyle habits facilitated by digital self-monitoring. This is represented by the quote from a nurse who works with patients using self-monitoring technology:

Inside the app, you get instructions and tasks about measuring, and the patient also has to fill in various questionnaires about lifestyle habits and other things, like mental health issues. So, we can assess them because many times, if someone is overweight and finds it difficult to exercise, we try to assist with that first. Or if someone smokes, we can connect them with smoking cessation support; we work a lot with lifestyle habits.

I25

#### Symptom Control Improvements

Digital self-monitoring may offer a practical platform for symptom management. By ensuring patients and health care professionals are on the same page regarding side effects and signs to watch out for, a more timely and effective intervention becomes possible. As a professional elaborated:

We will be helped because, hopefully, we get patients who remain longer in treatment, while at the same time educating both patients and doctors about the type of side effects to watch out for. Hopefully, they also get good control... they find out about it as soon as possible so they can address it...

I7

The “Improve” theme highlights tangible benefits and enhancements in health outcomes achieved through digital self-monitoring. With an emphasis on the proactive role of patients in their health journey, these tools potentially may foster positive shifts in lifestyle habits and more refined symptom management. The patient, armed with insights from self-monitoring, may consequently become an active participant in their care, leading to holistic improvements in their health and well-being. In addition, health care professionals may also actively engage patients in their own care, as presented below.

### Engage

#### Overview

Engagement, in the context of digital self-monitoring, underscores the participatory and interactive nature of health care, where patients actively contribute to their own process. Through the “Engage” theme, we delve into the mutual efforts of both patients and professionals in collaborative decision-making, planning, and the overall patient journey. This purpose enables patients to have a more significant say in their care. A potential challenge arises, however, from the question of who has the authority to make decisions and choose the route ahead, that is, determining who has the definitive say or the privilege of interpretation and decision-making based on the gathered data.

#### Coproducing Care

Highlighting the enhanced role of patients, an individual shared:

Patients who have been part of various projects have found it very positive. From the perspective that, in many ways, it has allowed them to feel they have gained greater influence in their own care and the opportunity to feel involved in their care.

I2

#### Interactive Planning

The interactive planning underscores the collaborative effort in crafting a patient’s care journey. A health care project leader with practical experience in implementing self-monitoring shared:

Together with the patient, [we] evaluate, document a plan, and order self-monitoring based on the existing needs.

I26

#### Agreements

The emphasis on agreements reiterates the essential connection, albeit remotely, between the patient and the health care provider. A national-level coordinator for self-monitoring expressed:

It’s not necessarily a person looking at these meetings daily, but there exists this handshake... an agreement between the patient and healthcare.

I29

This suggests the value of an online bond that replaces unnecessary physical interactions, ensuring continued connection.

#### Decision Support

Emphasizing the role of data in informed clinical decisions, an IT manager noted:

The foundation for meetings between the doctor and patient where I can see that you've slept poorly and didn't take your evening medication. This effect arose when you moved and ate this or that food. One can connect the feelings to much more data.

I2

#### Decision Authority

Highlighting the potential tug-of-war in data interpretation, a patient representative pointed out:

That is, healthcare often takes over, saying “this is what we should steer towards.” What healthcare professionals believe is most important for patients isn't always what patients think if you ask them.

I1

The “Engage” theme accentuates a collaborative landscape of health care, where digital self-monitoring tools enable patients to play a proactive role in their care journey. From coproducing care plans to reaching mutual agreements on treatment paths, the approach fosters greater patient autonomy. However, challenges arise, especially concerning who gets the final say in data interpretation, emphasizing the need for a balanced partnership between patients and health care professionals.

### Control

The “Control” purpose emerges out of the need to regulate certain aspects connected to the individual patient. A total of 2 subthemes emerge from this purpose: adherence to treatment or monitoring regimens and symptom control, which allows the patient to understand and manage their symptoms in real time.

#### Adherence

The role of digital self-monitoring in adherence directs attention toward the purpose that patients should follow their treatment plans more closely. This is especially significant when patients and health care providers agree upon certain monitoring parameters or when additional measurements are warranted based on specific symptoms. A representative from a tech company emphasized:

We might also decide that if you feel a certain way, we’d like you to take this measurement as well, like a symptom assessment, to tell us a bit about how you’re doing. Then you enter this routine, meaning that the patient weighs themselves with a frequency we've agreed upon.

I12

#### Symptom Control

Symptom control, on the other hand, enables patients to pinpoint and track symptoms over time, affording them a more nuanced understanding of their health status. Thus, digital self-monitoring may be an important tool for managing or alleviating the symptoms of a disease or condition rather than addressing its root cause. A patient representative mentioned:

This app, concerning well-being, allows me to self-measure if I feel pain somewhere. For instance, I can assess if I was swollen, where it hurts, whether it’s in the upper part of the leg, and I can measure this daily until it subsides.

I30

However, the ease of symptom control can vary depending on the specific health concern. An insight from a Parkinson’s patient underscores the challenges they face in symptom control. This sentiment is captured in the reflection :

I often say I have “diabetes envy.” By that, I mean when you live with type one diabetes, you have clear measurements to steer towards.

I1

This highlights the intricate relationship between symptom control and adherence. The ability to actively control and manage symptoms directly likely impacts a patient’s willingness and motivation to continue with self-monitoring.

### Evaluate: Clinical Decision-Making

At the crux of health care is the clinician-patient relationship. The role of digital self-monitoring in informing clinical decisions is perhaps the usual reason for its use, in the sense that it is grounded in up-to-date information about the patient’s health condition. Quote from an IT manager:

The collated data enhances the clinician-patient discourse. Observations like irregular sleep cycles or missed medication doses, when juxtaposed against dietary and activity patterns, can offer profound insights. This facilitates a more holistic understanding of the patient's health, enabling tailored interventions.

I15

Such feedback underlines the transformative potential of digital self-monitoring, elucidating its capability to fortify clinical decisions and, consequently, patient outcomes.

### Innovate

#### Overview

The “Innovate” purpose centers on the analysis and comprehension of data sourced from digital self-monitoring. This data is used at an aggregate level for research and the enhancement of health care delivery processes. While this technology is often used with the interests of clinical staff, administrative health care functions, and research and development enthusiasts in mind, it does not primarily stem from the patient’s viewpoint. From this perspective, such data becomes crucial, serving diverse needs, from advancing research and understanding organizational processes and outcomes to aiding clinical decisions. This objective focuses primarily on self-monitoring data at the group level rather than connecting it directly to the individual patient. The 2 variations of this theme are outlined below.

#### Research

The scope of digital self-monitoring is used as a tool in clinical research. By harnessing data from these tools, researchers can glean real-world insights into various medical phenomena:

Self-tracking inherently serves as a potent method for data collection, aligning seamlessly with conventional clinical research.

I1

Such testimonials underscore the expanding role of digital self-monitoring in the realm of research, fostering the development of evidence-based methods.

#### Organizational Effectiveness

Digital self-monitoring also has the potential to contribute information that supports the evaluation of organizational effectiveness. Professionals within the sector have underscored its ability to induce marked improvements in how health care processes and systems operate. A pharmaceutical representative said:

When examining the data, it’s evident that emergency hospital admissions have decreased by 53% over a 2-year period, a statistic that speaks volumes. However, contrasting figures emerge when comparing with other clinics, some of which report marginally lesser reductions.

I19

This observation highlights that digital self-monitoring can provide health care organizations with valuable information to improve their operations and patient outcomes at a group level.

### Generate

The purpose of “Generate” in digital health care self-monitoring is rooted in the use of existing digital infrastructure, which serves as both the foundation and the motorway for further advancements. Digital self-monitoring leverages this underlying architecture, using it not just for immediate patient-centric needs but also as a platform for broader health care innovations, such as integrating new forms of self-monitoring based on already existing structures. Just as motorways accelerate transportation expansion, this digital backbone ensures the system is adaptive and scalable for future demands. This is exemplified by the following quote from a MedTech representative:

You know, with all this tech and digital self-monitoring in healthcare, it’s like we’ve built a motorway that’s open for other new ideas and journeys. Kind of like how those AI things learn from what they know and just keep getting smarter.

I11

The data derived from self-monitoring, while primarily catering to clinical and administrative functions, can also be used to understand broader health care dynamics. Such data, enabled by a robust infrastructure, becomes a potent tool in pioneering research, refining organizational processes, and shaping clinical decisions.

Ultimately, this “Generate” objective goes beyond individual patient data, emphasizing the collective potential harnessed through this motorway of digital infrastructure, which continues to evolve and drive the health care sector forward with agility and foresight, as exemplified by the following quote:

…but we must cooperate for it to become something, and it has to be quite large and broad efforts for it to make any sense. I would say that we are still perhaps operating across the entire spectrum, but if you ask me, I think that these, these bigger things that can be done together.

I27

### Summary and Synthesis of Results

**Figure 1:**
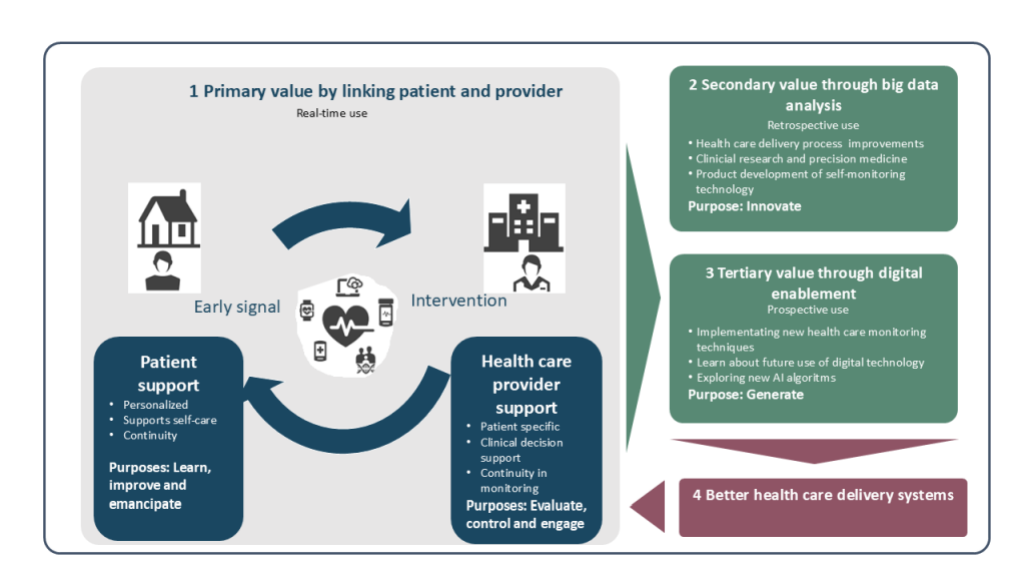
The 3 categories of purposes for using digital health care self-monitoring. AI: artificial intelligence.

First, digital health care self-monitoring seeks to emancipate patients, granting them more autonomy over their health data and decisions. This empowerment can directly lead to improvements in the patient’s health. As health care providers delve deeper into the data, they can learn more about individual patient health behaviors, which in turn allows them to engage patients more profoundly in their own health journey. Second, the technology can provide means to control adherence and symptom management more effectively. A foundational purpose is to evaluate patients’ health parameters. Third, there are also purposes that stretch beyond immediate patient care and focus on innovating within health care processes based on aggregated data from the established infrastructure of digital health care self-monitoring, offering insights into health trends and outcomes. Thus, the 8 identified purposes can be clustered into 3 main categories relating to the way in which they contribute to value: (1) improving the link between patient and provider, (2) enhancing existing care through insights gained from analyzing big data collected through digital health care self-monitoring, and (3) value encompassing broader systemic impacts (Figure 1).

#### Linking Patient With Provider

##### For the Patient

The purposes of “Learn,” “Improve,” and “Emancipate” underscore the essential role of digital tools in empowering patients with knowledge, aiding their self-improvement journey, and giving them a sense of autonomy in their health journey.

##### For the Provider

“Evaluate,” “Control,” and “Engage” are geared toward health care providers, focusing on the assessment of patient health, maintaining oversight, and fostering deeper patient engagement.

#### Big Data Analysis—Create Knowledge From Patient-Generated Data

“Innovate” underscores the significance of harnessing big data. This purpose emphasizes breakthroughs, insights, and new methodologies that arise from analyzing vast datasets in health care.

#### Digital Enablement—Using the Infrastructure of Digital Health Care Self-Monitoring to Identify New Opportunities

“Generate” highlights the ways in which digital technologies can create and open up new possibilities in health care, exploiting the potential of digital health care self-monitoring infrastructures to produce improved health outcomes.

## Discussion

### Background

Digital health care self-monitoring creates high expectations, and stakes are notably elevated. However, broad adoption is challenging. The challenge is not just introducing new technology but ensuring an organization’s readiness to integrate it [26], that is, moving beyond a pilot state. Implementing technical innovations in health care is known to have a high rate of failure, often related to different aspects of complexity not noticed during the development process. Such aspects involve both the technical device itself and the intended value for intended users, relevant regulations, and organizational readiness. Thus, evaluating the purpose of digital health care self-monitoring before the start of a project on digital health care self-monitoring likely enhances the likelihood of moving beyond the pilot stage.

### Principal Findings

This study suggests 8 primary purposes of using digital health care self-monitoring: emancipate (granting patients autonomy), learn (understanding patient symptoms and behaviors), improve (enhancing patient’s health), engage (deepening patient involvement), control (managing adherence and symptoms), evaluate (assessing health parameters), innovate (improving interventions, health care processes, and technology), and generate (driving new initiatives). The 8 purposes, stemming from varied origins such as research and development interests, clinical interactions with individual patients, and the notion of the patient as a competent and autonomous individual, can sometimes be at odds with each other. As an example, the “Innovative” purpose emphasizes the collection and analysis of group-level data. This approach, while essential for the formulation of standardized treatment protocols, often informs the design of digital tools that prioritize population-based insights. On the other hand, “Control” is distinctly oriented toward individual patient outcomes, underscoring symptom management and adherence to prescribed treatments. The integration of these contrasting purposes can entail challenges. A system predominantly oriented toward evaluation, for instance, might not adequately account for the diverse and intricate nuances associated with individual patient care.

### Comparison With Previous Work

In terms of theoretical implications, previous research has called for improving the conditions within the health care ecosystem to promote better health outcomes and high-value health care [26]. When considering the balance between clinical research and patient interests, the design disparities become salient. An evaluative framework designed to uphold evidence-based practices drawn from broad datasets may inadvertently be at odds with features aiming to foster “Learning,” “Improvement,” and “Emancipation.” These latter purposes are geared toward empowering patients and making them more informed and autonomous. However, if organizations strive to better support health literacy [16] by promoting digital health care self-monitoring, patients might not only gain a higher degree of independence and health-related quality of life but also move quicker through the health care system, thus demanding less help and attention. Consequently, striking a design balance that both respects rigorous empirical evidence and accommodates the patient’s evolving agency is a critical challenge. Furthermore, such design recommendations can be scrutinized from the perspective of both inter- and intraorganizational learning [27], hence providing an evaluation model accounting for the satisfaction of users as well as cost [16].

While previous research highlights the difficulty of transforming data measured and reported by patients into useful knowledge [26], this study found that this challenge might arise from the lack of inclusion of multiple stakeholders at the outset, as there are differences in purposes for digital health care self-monitoring between the stakeholders—some of which might even be contradictory. The 2 examples of such contradictions are (1) a patient might be motivated by “Learning” about their own health status, while the MedTech company is motivated by “Innovating” technology through extracting patient data. The solution to this problem lies in balancing these different purposes while still ensuring safety and quality in health care, and (2) health care providers might focus on “control” and thus prioritize features such as monitoring and alerts to ensure treatment adherence. At the same time, patients focus on “engagement” through interactive user experience. Integrating these distinct modalities into a singular digital tool is challenging: an interface that leans too heavily toward “control” might diminish user engagement, while an interface that excessively prioritizes “engagement” might risk sidelining essential clinical guidance. While previous research suggests that technology might contribute to patient-centered care [26], there is a need for a coordinated and systemic approach to successfully use and adopt digital health care self-monitoring technology—while still maintaining patient-centered care. Such an approach involves creating awareness of the diverse purposes among different stakeholders; thus, it is essential for all stakeholders to understand “why” digital health care self-monitoring is important for all stakeholders and how the “why” differs among the stakeholders. Once the stakeholders involved are aware of each other’s underlying purposes, conflicting tensions can potentially be resolved if considered in the design or improvement of digital health care self-monitoring tools.

### Strengths and Limitations

This study’s results can help define the why and thereby find better forms of evaluation from the expected outcome. Using the 8 categories of purpose as a starting point, and even hierarchically ordering them, can enable a clearer purpose and expected value. While pilot tests assess technology efficacy, a more holistic view that includes the purposes and needs of various stakeholders is often absent during broader implementation. Transitioning from innovation to implementation is to move from focusing solely on technology to ensuring it aligns with existing or envisioned operations. This study has two main limitations. First, the study is limited to one specific national context in which health care is government-funded, and thus, certain purposes might differ in a health care system with another funding scheme and other stakeholders. Second, the study is of an explorative nature and, thus, limited in its possibility to draw conclusions about the direct effects of focusing on the identified purposes of the outcome of the implementation of digital health care self-monitoring.

### Future Directions

In summary, future research could focus on how knowledge about the different purposes of digital health care self-monitoring can be transferred into design recommendations for digital innovations. If the purpose is clear, complexity is noticed, and the primary value-receiver is defined, a more adequate design can be recommended early on in the process, hence avoiding pitfalls along the way. Another potential avenue for future research is to move outside the national context of Sweden, characterized by a government-funded health care system, to a national context with another funding scheme. Finally, quantitative studies on the purposes identified and how consideration of them could impact the implementation of digital health care self-monitoring would be of interest for future research.

### Conclusions

In conclusion, as digital health care self-monitoring tools evolve within the health care sector, the inherent design and implementation challenges they present underscore the need for a nuanced approach to encompass the needs and purposes of various stakeholders. In this paper, 8 different purposes for using digital health care self-monitoring are empirically derived: “Learn,” “Improve,” “Emancipate,” “Evaluate,” “Control,” “Engage,” “Innovate,” and “Generate.” Furthermore, these purposes point to 3 categories of purposes: enhancing the link between patient and health care provider (primary value), creating knowledge from patient-generated data by big data analytics to improve care (secondary value), and using digital health care self-monitoring to enable identification of new possibilities to re-design or innovate care processes (tertiary value).

## Appendix

Multimedia Appendix 1 Interview guide.

Multimedia Appendix 2 Quotes.

## Data Availability

The datasets used and analyzed during this study are available from the corresponding author on reasonable request.
