# Supplementary material for: Identification and Categorization of the Distinct Purposes Underpinning the Use of Digital Health Care Self-Monitoring: Qualitative Study of Stakeholders in the Health Care Ecosystem
Source: J Med Internet Res. 2025 Apr 3;27:e58264. doi: 10.2196/58264 (PMC12006770; doi:10.2196/58264)
Supplement: Multimedia Appendix 1 [file jmir_v27i1e58264_app1.docx]

**Appendix I**

**Interview Guide 1: Micro-Level Perspectives on Digital Healthcare Self-Monitoring**

*(For healthcare professionals and advocacy groups)*

**General Questions**

1. What do you know about digital self-monitoring in healthcare?
2. How do you view self-monitoring in the healthcare system?
3. What is the greatest benefit of self-monitoring, and what are your key takeaways?
4. What challenges do you see in implementing self-monitoring?

**Specific Questions for Micro-Level Participants**

**Work Approach & Processes**
5. How has the process or working method been structured when implementing digital self-monitoring?
6. Which actors have been involved in your work with digital self-monitoring?
7. Have you used any specific methods or models to integrate digital self-monitoring into your work?

**Patient Experience & Impact**
8. How was self-monitoring perceived and experienced by your patients?
9. How have you used the information from self-monitoring to assist your patients?
10. How do you engage and motivate your patients to continue using self-monitoring tools over time?
11. What role does feedback play in the self-monitoring process? How and when do you provide feedback to your patients? (e.g., digitally, via email, phone calls, etc.)
12. What are the main reasons why patients or other actors might discontinue their participation in self-monitoring?

**Ethical & Systemic Considerations**
13. Have you encountered ethical concerns related to digital self-monitoring? If so, what were they?
14. How does digital self-monitoring fit into the overall healthcare ecosystem?
15. How do you see the future of digital self-monitoring in healthcare?

**Interview Guide 2: Macro-Level Perspectives on Digital Healthcare Self-Monitoring**

*(For policy-makers, pharmaceutical companies, tech firms, IT managers, software developers, and administrative managers)*

**General Questions**

1. What do you know about digital self-monitoring in healthcare?
2. How do you view self-monitoring in the healthcare system?
3. What is the greatest benefit of self-monitoring, and what are your key takeaways?
4. What challenges do you see in implementing self-monitoring?

**Specific Questions for Macro-Level Participants**

**Systemic & Strategic Perspectives**
5. How does digital self-monitoring fit within your organization’s strategic priorities?
6. What role does your organization play in the development, implementation, or regulation of digital self-monitoring?
7. How does your organization collaborate with other stakeholders (e.g., healthcare providers, technology developers, regulators) to advance digital self-monitoring?

**Barriers & Opportunities**
8. What are the biggest barriers to the adoption and scaling of digital self-monitoring tools?
9. What are the key incentives for different stakeholders (healthcare providers, policymakers, tech developers) to support self-monitoring solutions?
10. How do financial, regulatory, or technological factors influence the success of digital self-monitoring initiatives?

**Data, Ethics & Governance**
11. How does your organization address data privacy and security concerns related to digital self-monitoring?
12. Have you encountered ethical concerns related to self-monitoring? If so, what were they?
13. How can policies and regulations better support the responsible use of digital self-monitoring in healthcare?

**Future Perspectives**
14. How do you see the future of digital self-monitoring in healthcare?
15. What steps do you believe are necessary to further integrate digital self-monitoring into mainstream healthcare systems?
