# Supplementary material for: Identification and Categorization of the Distinct Purposes Underpinning the Use of Digital Health Care Self-Monitoring: Qualitative Study of Stakeholders in the Health Care Ecosystem
Source: J Med Internet Res. 2025 Apr 3;27:e58264. doi: 10.2196/58264 (PMC12006770; doi:10.2196/58264)
Supplement: Multimedia Appendix 2 [file jmir_v27i1e58264_app2.docx]

**Appendix II – Quotes illustrating the purposes for digital healthcare self-monitoring**

| **Purpose** | **Illustrative Quote** | **Interviewee identifier (see Table 1)** |
| --- | --- | --- |
| **Emancipate** | "If [a patient] could just live [his or her] life without having to adapt to the opening hours and locations of healthcare, that’s what I desire. [you] want to live as normally as [you] can despite the illness, and this technology makes that feasible." | I10 |
|  | "The team that manages my disease is always accessible in some way." | I30 |
|  | "The healthcare system always acts as a backup and proactively intervenes if health metrics start to change in a way that might necessitate treatment adjustments." | I13 |
|  | "She no longer feels the dread of death, and doesn't always feel the need to be close to her healthcare centre." | I31 |
| **Learn** | "...to rate their own sense of quality of life during treatment and then also... the idea was to take more control over one's daily life so that patterns related to what one ate or how one moved could be discerned. If one felt unwell one day, why? How did I live that day compared to days I felt well?" | I2 |
|  | "When a measurement exceeds a specific threshold, what kind of self-care recommendations should be provided?" | I6 |
|  | "If you manage your diet, exercise, sleep well, and take your medication correctly... then you can see a certain pattern statistically. Then it becomes interesting to ask: 'How did you feel when you woke up this morning? How did you think your day would be compared to how your day went when you go to bed in the evening?'" | I26 |
|  | "I track when I have a specific question to answer. I believe in doing it for a short period... because when I feel good enough, I don't want to spend time tracking. I want to live my life..." | I9 |
| **Improve** | "Inside the app, you get instructions and tasks about measuring, and the patient also has to fill in various questionnaires about lifestyle habits and other things, like mental health issues. So, we can assess them because many times, if someone is overweight and finds it difficult to exercise, we try to assist with that first. Or if someone smokes, we can connect them with smoking cessation support; we work a lot with lifestyle habits." | I25 |
|  | "We will be helped because, hopefully, we get patients who remain longer in treatment, while at the same time educating both patients and doctors about the type of side effects to watch out for. Hopefully, they also get good control... they find out about it as soon as possible so they can address it..." | I7 |
| **Engage** | "Patients, who have been part of various projects, have found it very positive. From the perspective that, in many ways, it has allowed them to feel they have gained greater influence in their own care and the opportunity to feel involved in their care." | I2 |
|  | "Together with the patient, [we] evaluate, document a plan, and order self-monitoring based on the existing needs." | I26 |
|  | "It's not necessarily a person looking at these meetings daily, but there exists this handshake... an agreement between the patient and healthcare." | I29 |
|  | "The foundation for meetings between the doctor and patient where I can see that you've slept poorly and didn't take your evening medication. This effect arose when you moved and ate this or that food. One can connect the feelings to much more data." | I2 |
|  | "That is, healthcare often takes over, saying 'this is what we should steer towards.' What healthcare professionals believe is most important for patients isn't always what patients think if you ask them." | I1 |
| **Control** | "We might also decide that if you feel a certain way, we'd like you to take this measurement as well, like a symptom assessment, to tell us a bit about how you're doing. Then you enter this routine, meaning that the patient weighs themselves with a frequency we've agreed upon." | I12 |
|  | "This app, concerning well-being, allows me to self-measure if I feel pain somewhere. For instance, I can assess if I was swollen, where it hurts, whether it's in the upper part of the leg, and I can measure this daily until it subsides." | I30 |
|  | "I often say I have 'diabetes envy'. By that, I mean when you live with type one diabetes, you have clear measurements to steer towards." | I1 |
| **Evaluate** | "The collated data enhances the clinician-patient discourse. Observations like irregular sleep cycles or missed medication doses, when juxtaposed against dietary and activity patterns, can offer profound insights. This facilitates a more holistic understanding of the patient's health, enabling tailored interventions." | I15 |
| **Innovate** | "Self-tracking inherently serves as a potent method for data collection, aligning seamlessly with conventional clinical research." | I1 |
|  | "When examining the data, it's evident that emergency hospital admissions have decreased by 53% over a 2-year period, a statistic that speaks volumes. However, contrasting figures emerge when comparing with other clinics, some of which report marginally lesser reductions." | I19 |
| **Generate** | "You know, with all this tech and digital self-monitoring in healthcare, it's like we've built a motorway that's open for other new ideas and journeys. Kind of like how those AI things learn from what they know and just keep getting smarter." | I11 |
|  | “but we must cooperate for it to become something and it has to be quite large and broad efforts for it to make any sense. I would say that we are still perhaps operating across the entire spectrum, but if you ask me, I think that these, these bigger things that can be done together." | I27 |
